# The trigeminal function questionnaire (TriFunQ): a tool for clinical and research use

**DOI:** 10.1093/chemse/bjag013

**Published:** 2026-05-14

**Authors:** Wiktoria Jędryczka, Susanne Weise, Anabell Bormann, Thomas Hummel

**Affiliations:** Smell and Taste Clinic, Department of Otorhinolaryngology, University Hospital Carl Gustav Carus, TU Dresden, Dresden, Germany; Institute of Psychology, University of Wroclaw, Wroclaw, Poland; Smell and Taste Clinic, Department of Otorhinolaryngology, University Hospital Carl Gustav Carus, TU Dresden, Dresden, Germany; Smell and Taste Clinic, Department of Otorhinolaryngology, University Hospital Carl Gustav Carus, TU Dresden, Dresden, Germany; Smell and Taste Clinic, Department of Otorhinolaryngology, University Hospital Carl Gustav Carus, TU Dresden, Dresden, Germany; Department of Nutritional Science and Food Management, Ewha Womans University, Seodaemun-gu, Seoul, Korea

**Keywords:** smell, olfaction, trigeminal stimulation, trigeminal system

## Abstract

The purpose of the study was to develop a self-reporting measure of trigeminal function, addressing the need for the participants’ self-perception of comfort and sensitivity related to trigeminal sensations. A 15-item questionnaire was designed and tested on the sample of *n* = 1,001 participants from the general population in Dresden, Germany, aged from 18 to 86. For validation included were ratings of nasal trigeminal stimuli using ammonium, lateralization of eucalyptol as a measure of trigeminal sensitivity, odor identification, and self-ratings of nasal airflow and olfactory function. Based on factor analyses, the initially intended structure of the questionnaire was revised to improve the tool's overall psychometric quality. Originally intended structure was not well supported. Exploratory factor analysis based on a scree plot indicated 1-factor solution, with the final version comprising 8 carefully selected items, providing the best fit. The questionnaire presented good reliability (Cronbach's *α* = 0.74) and satisfying validity, based on correlations with other self-reporting measures and group comparisons. Invariance analysis across gender suggested the use of separate norms for men and women. The trigeminal function questionnaire is a brief, reliable and valid measure for assessing self-perception of trigeminal function.

## 1. Introduction

Olfactory sensations result from complex activations of both the olfactory and trigeminal systems, which can be further modulated by visual, auditory, and emotional inputs. The trigeminal system, responsive to mechanical, thermal, chemical and nociceptive stimuli, plays a crucial role in olfactory perception. Further, the trigeminal system mediates the perception of nasal airflow. Many odorants activate both the olfactory and the trigeminal system, which are interlinked at both peripheral and central levels (Hummel and Frasnelli 2019).

When it comes to pathological conditions such as olfactory dysfunction (OD), diagnostical tools are necessary to measure the functionality. The prevalence of OD are ∼20%, with 5% being anosmic (Brämerson et al. 2004; Landis et al. 2004; Whitcroft et al. 2023), which can lead to severe consequences, including reduced enjoyment of food, risks for household accidents (Santos et al. 2004), insecurity in social situations (Schaal et al. 1980; Croy et al. 2013; Schäfer et al. 2024), increased risk for depression (Croy et al. 2014; Croy and Hummel 2017), and an increased 5-yr mortality rate (Pang et al. 2022). Further, in patients with OD, there is an interaction between the olfactory and trigeminal system, as a reduced trigeminal function was detected in patients with OD (Gudziol et al. 2001; Ren et al. 2012). In addition, trigeminal function declines with age (Hummel et al. 1998, 2003; Frasnelli and Hummel 2003) and it is also influenced by nasal anatomy (Konstantinidis et al. 2010).

To assess intranasal trigeminal sensitivity, various psychophysical tests have been developed (Garefis et al. 2024). These include the lateralization task, which exploits the ability to localize trigeminal stimuli to 1 nostril (Frasnelli et al. 2011). A reduced ability to correctly identify the stimulated side shows an impaired trigeminal function (Mai et al. 2025b). Further, intensity ratings for chemical stimuli such as the AmmoLa can be used to quantify the trigeminal function with ratings below the 10th percentile respectively below 15% on the visual analogue scale for the intensity ratings indicate trigeminal and OD (Sekine et al. 2022). Trigeminal Sticks test (Burghart Medical Technology, Wedel, Germany), modeled after the Sniffin’ Sticks test for olfactory assessment, uses felt-tip pens filled with trigeminal compounds that activate different trigeminal receptors to measure trigeminal threshold, discrimination, and identification (Huart et al. 2019). In addition, the detection threshold for carbon dioxide (CO_2_) can measure trigeminal function (Hummel et al. 2016). Trigeminal tests present some difficulties, as they involve stimulation of different modalities of the trigeminal nerve (e.g. chemical or electrical) and different receptor pathways, which may engage nonoverlapping functional characteristics (Hummel and Frasnelli 2019), except for the Trigeminal Sticks test as different receptors are addressed. Carbon dioxide (CO_2_) primarily activates acid-sensitive ion channels, transient receptor potential vanilloid 1 (TRPV1) receptor (Wang et al. 2010), and transient receptor potential (TRPA1 Mai et al. 2025), menthol activates transient receptor potential melastatin 8 channels (McKemy et al. 2002), and ammonia stimulates TRPA1 and TRPV1 among (Dhaka et al. 2009). These differences may lead to varying perceptual and physiological responses, complicating the interpretation and standardization of trigeminal function testing. In summary, current psychophysical tests of trigeminal function are limited by a lack of normative data (except for lateralization test), restricted commercial availability (except for AmmoLa), and variable testing times. Moreover, they show inconsistent associations with sex differences and age-related decline (Mai et al. 2025).

However, more objective approaches to quantify trigeminal function exist, such as electrophysiological techniques that assess peripheral responses through negative mucosa potentials (Kobal 1985) and central processing via event-related potentials (Hummel and Frasnelli 2019). Further, magnetoencephalography can be used to analyze central processing of trigeminal stimulation. However, these methods are primarily used for research purposes and selected clinical cases. While several validated psychophysical tests exist to assess orthonasal olfactory function such as Sniffin’ sticks (Hummel et al. 1997; Oleszkiewicz et al. 2019) and University of Pennsylvania Smell Identification Test (Doty et al. 1984) only the lateralization task has normative data as a psychophysical trigeminal test (Mai et al. 2025b), which needs to be validated for patients with trigeminal dysfunction. Interestingly, the link between the olfactory and the trigeminal test can be seen in the psychophysical trigeminal test with for example lower AmmoLa ratings (Sekine et al. 2022) and lower performance in the lateralization test for patients with OD (Mai et al. 2025b). Previous studies showed, that there is a low correlation between subjective olfactory function and psychophysical olfactory testing (Landis et al. 2003). To improve the standardization of subjective ratings, the questionnaire for individual significance of olfaction proved to be a valuable tool for reliably assessing individual perceptions of olfactory function (Croy et al. 2010).

To date, no standardized instrument exists to assess subjective trigeminal function in a time-efficient and practical manner suitable for use in clinical practice and research contexts. A questionnaire could therefore be helpful in the clinical context to characterize the trigeminal activation in everyday life. The aim of this study was to evaluate and refine a questionnaire designed to characterize the intranasal trigeminal function.

## 2. Materials and methods

### 2.1 Participants

This prospective study was conducted according to the Declaration of Helsinki in a public museum “Deutsches Hygiene-Museum” in Dresden, Germany. The study had been approved by the local ethics board (EK-343082023). All participants provided written informed consent.

The inclusion criteria were age of 18 yr and older, and absence of a significant disease that could affect the sense of smell (e.g. Parkinson's disease, significant renal disorder). Exclusion criteria included pregnancy and breastfeeding. A total of 1,001 participants (aged 18 to 86 yr, mean 44.2 ± 16.8 yr, *n* = 598 [59.74%] women, *n* = 396 [39.6%] men and *n* = 7 [0.66%] of those who declined to identify as men or women) were recruited for the study.

### 2.2 Questionnaire

The trigeminal function questionnaire (TriFunQ) initially consisted of 15 four-scaled items, phrased as a personal statement. The participants were asked to indicate how much they agree with the statements, from 0—“I totally disagree” to 3—“I totally agree.”

Similar to the questionnaire of individual significance of olfaction, the present instrument was designed to assess different domains related to trigeminal function, including Application (*n* = 8), Consequence (*n* = 3), and Association (*n* = 4), which were intended to serve as subscales in the subsequent analysis. Additionally, the questionnaire addressed various modalities of trigeminal stimulation, such as thermal (*n* = 3), chemical (*n* = 11), and mechanical stimuli (*n* = 2), although there was marginal overlap between questions.

### 2.3 Procedure

Following comprehensive study information, participants completed a standardized questionnaire covering medical history, including potential conditions affecting olfactory function, self-ratings of the olfactory function on a scale from 0 to 100 (0 = no olfactory function, 100 = excellent olfactory function) and the nasal airflow for both nostrils, individually and combined, on a subjective scale from 0 to 100 (0 = completely blocked, 100 = very wide nasal passages), as well as the TriFunQ. All participants underwent a screening test for olfactory function using the “Sniffin’ Sticks” (Burghart Messtechnik GmbH, Holm, Germany) with the 3 odorants: banana, cinnamon and fish (Lötsch et al. 2016).

Further all participants underwent the intensity rating of the AmmoLa stick (AmmoLa, Devesa Dr. Reingraber GmbH, Muggensturm, Germany), which contains a mixture of lavender and the pungent ammonium. Reduced intensity ratings (<10th percentile; visual analogue scale [VAS] of 15%) are associated with OD and reduced trigeminal function (Sekine et al. 2022).

In a subset of participants, lateralization testing was conducted (*n* = 548) using 2 identical squeezable bottles, which were simultaneously compressed to release an equal airstream into both nostrils. Only one bottle contained an odorant (menthol, 50% v/v diluted in 1,2-propanediol). Participants, wearing a facial mask to eliminate visual input, were asked to detect the nostril exposed to the odor stimulus using a forced-choice paradigm (Frasnelli et al. 2011) in a 20-trial task. Impaired detection of the correct side is indicative of reduced trigeminal function (Mai et al. 2025b).

Each of these procedures was conducted according to standardized protocols in temperature-controlled rooms (21 to 22 °C) to ensure consistency across measurements. Participants were instructed to abstain from using perfumes, consuming food or beverages (except water), smoking, or chewing gum for at least 1 h prior to the assessment to minimize confounding factors.

### 2.4 Statistical analysis

Statistical analyses were performed using RStudio (version R 4.3.1, with *psych* [Revelle 2025], *lavaan* [Rosseel 2012], *ggplot2* [Wickham et al. 2025], *effectsize* [Ben-Shachar et al. 2025], *rstatix* [Kassambara 2019], *dplyr* [Wickham et al. 2023], and *tidyr* [Wickham et al. 2014] packages). Descriptive statistics are presented in Table 1.

**Table 1:** Descriptive statistics.

Variable	*N*	Min	Max	*M* (SD)
Trigeminal questionnaire—original score	1,001	0	46	21.21
Trigeminal questionnaire—8 item score	963	0	24	9.13 (4.28)
Lateralization	548	4	20	14.44 (3.26)
Identification	1,001	0	3	2.63 (0.60)
AmmoLa	1,000	0	10.2	9.13 (1.26)
Nasal sensitivity to stinging/burning	1,001	0	4	2.34 (0.86)
Evaluation of nasal airflow	1,000	0	10.2	7.02 (2.05)
Self-rated olfactory function	997	0	10.2	7.22 (2.08)
Age	991	18	86	44.19 (16.81)

The sample consisted of *n* = 598 (59.74%) females, *n* = 396 (39.6%) males and *n* = 7 (0.66%) participants who did not share information about their sex. One hundred and seventy-four (17.4%) participants had reported having some medical conditions, including sinusitis, allergies, chronic rhinosinusitis [CRS] surgery, or others, while *n* = 827 (82.6%) did not report any. Most participants (*n* = 883, 83.5%) had recovered from COVID-19 infection, and *n* = 165 (16.5%) were never infected.

## 3. Results

To structure the trigeminal questionnaire, confirmatory factor analysis (CFA) was conducted. At first, the questionnaire was intended to include 3 subscales—Association, Application, and Consequence of the activation of trigeminal function similar to a Questionnaire on the Significance of Olfaction (Croy et al. 2010). Association would refer to emotional and memorial response, Application would refer to the actual use of trigeminal function reported by the participant and Consequence would refer to how trigeminal activation influences one's behavior. The intended list of factors is featured in Table 2. For the further analysis, to avoid biasing the results, only data from participants who have answered all 15 questions of the questionnaire was taken into consideration (*n* = 936). After establishing the questionnaire, participants who provided answers to the selected questions were, once again, included to the analysis (*n* = 963).

**Table 2:** Initial items of trigeminal questionnaire.

Item	Intended factor	Modality
Pungent or burning smells like smoke, vinegar or nail polish remover evoke strong emotions in me.	Association	Chemical
When I chew a fresh mint gum, I feel like I can breathe better through my nose.	Application	Chemical and nasal patency (airflow) = mechanical
I avoid carbonated drinks because you feel a stinging sensation in your nose when you burp.	Consequence	Chemical
I consider my nasal breathing to be very good.	Application	Nasal patency (airflow) = mechanical, thermal
I do not like going to a sauna because I find hot air in my nose very burning.	Consequence	Thermal
When I cut onions, my eyes water a lot.	Application	Chemical
Pungent or burning smells make me cough or sneeze.	Application	Chemical
I avoid burning, pungent smells (e.g. ammonia or chlorine).	Consequence	Chemical
When I smell something pungent or stinging, I panic and think of situations in which something similar has happened to me.	Association	Chemical
In winter, the cold air in my nose is extremely unpleasant.	Association	Thermal
When I eat horseradish, I find the stinging in my nose particularly annoying.	Association	Chemical
Burning or pungent smells can cause unpleasant sensations/pain in my face.	Application	Chemical and nociception
I am aware and intensely aware of the carbonation in drinks.	Application	Chemical
When it comes to slightly tickly or pungent smells, my nose is much more sensitive than other people's noses.	Application	Chemical
I only use toothpaste with a very mild mint smell.	Application	Chemical

Contrary to expectations, CFA showed poor fit of the model with 3 intended factors (*CFI* = 0.921, *TLI* = 0.905, *RMSEA* = 0.042), suggesting that 3 factors are not optimal.

**Figure 1:**
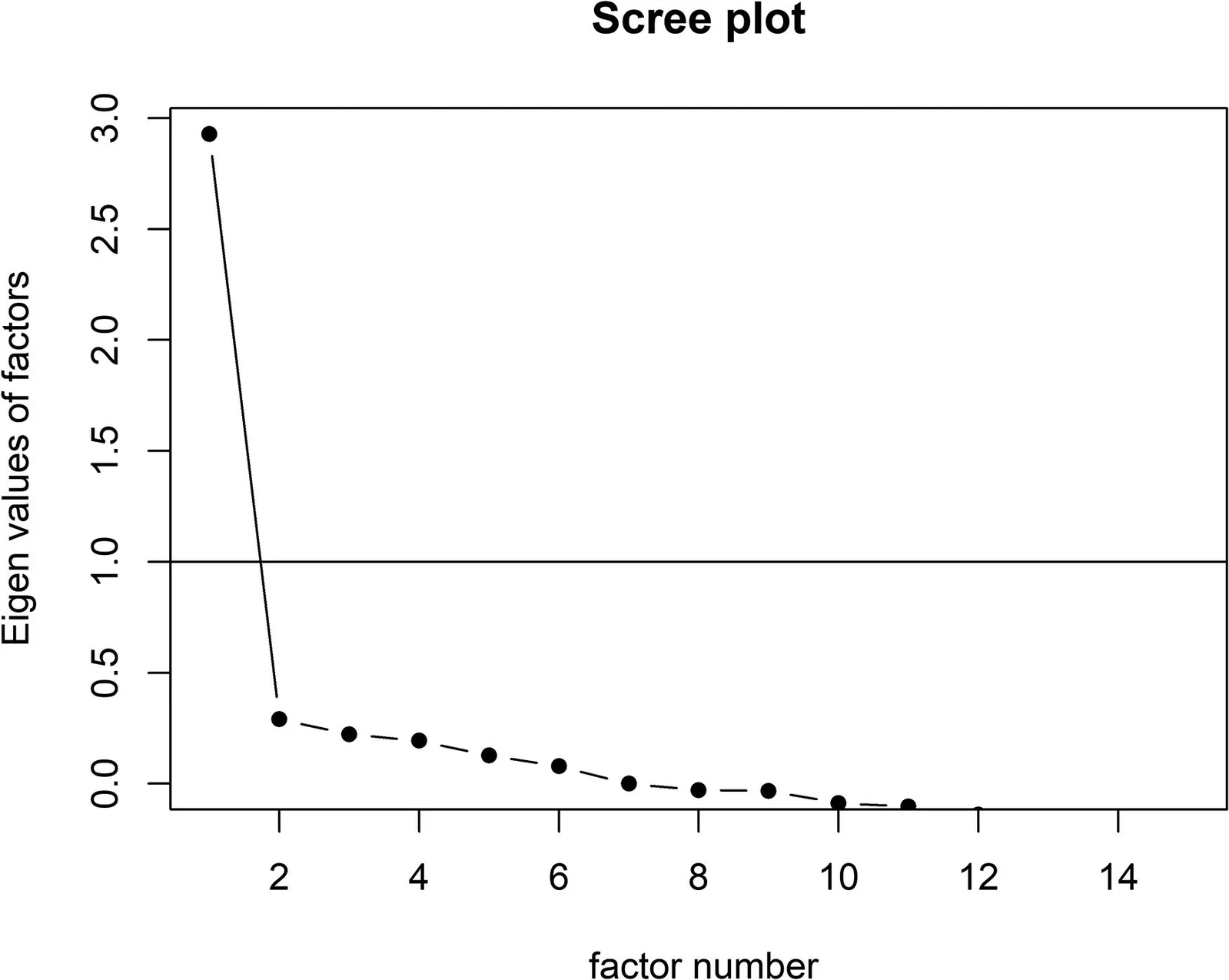
Scree plot of possible factors of the trigeminal questionnaire. The analysis indicated that only one factor contributed to the results.

The next step was then to explore data to see what number of factors can be observed. The scree plot (Fig. 1) indicated that only one factor was justified.

As indicated by the scree plot, in the next step all items were included as 1 factor—and the CFA was repeated. However, indices of fit were still poor (*CFI* = 0.917, *TLI* = 0.903, *RMSEA* = 0.042). Factor loadings of items were then analyzed to identify the utility of particular items to the model (items achieving factor loading of 0.45 or more were further included). This way, 8 items were selected and analyzed as a separate model. CFA showed fine indices of fit (*CFI* = 0.969, *TLI* = 0.957, *RMSEA* = 0.043). Standardized factor loadings of both models are displayed in the Table 3.

**Table 3:** Factor loadings in various models.

Item	Standardized factor loading (15-item model)	Standardized factor loading (8-item model)
Pungent or burning smells like smoke, vinegar or nail polish remover evoke strong emotions in me.	0.422	…
When I chew a fresh mint gum, I feel like I can breathe better through my nose.	0.212	…
I avoid carbonated drinks because you feel a stinging sensation in your nose when you burp.	0.450	0.404
I consider my nasal breathing to be very good.	0.065	…
I do not like going to a sauna because I find hot air in my nose very burning.	0.335	…
When I cut onions, my eyes water a lot.	0.259	…
Pungent or burning smells make me cough or sneeze.	0.536	0.561
I avoid burning, pungent smells (e.g. ammonia or chlorine).	0.514	0.523
When I smell something pungent or stinging, I panic and think of situations in which something similar has happened to me.	0.589	0.611
In winter, the cold air in my nose is extremely unpleasant.	0.455	0.444
When I eat horseradish, I find the stinging in my nose particularly annoying.	0.452	0.455
Burning or pungent smells can cause unpleasant sensations/pain in my face.	0.572	0.597
I am aware and intensely aware of the carbonation in drinks.	0.399	…
When it comes to slightly tickly or pungent smells, my nose is much more sensitive than other people's noses.	0.577	0.547
I only use toothpaste with a very mild mint smell.	0.435	…

The 8-item model presented good reliability (standardized Cronbach's *α* = 0.741). Participants obtained results from 0 to 24, with average scores of *M* = 9.07 ± 4.29.

### 3.1 Validity check

**Figure 2:**
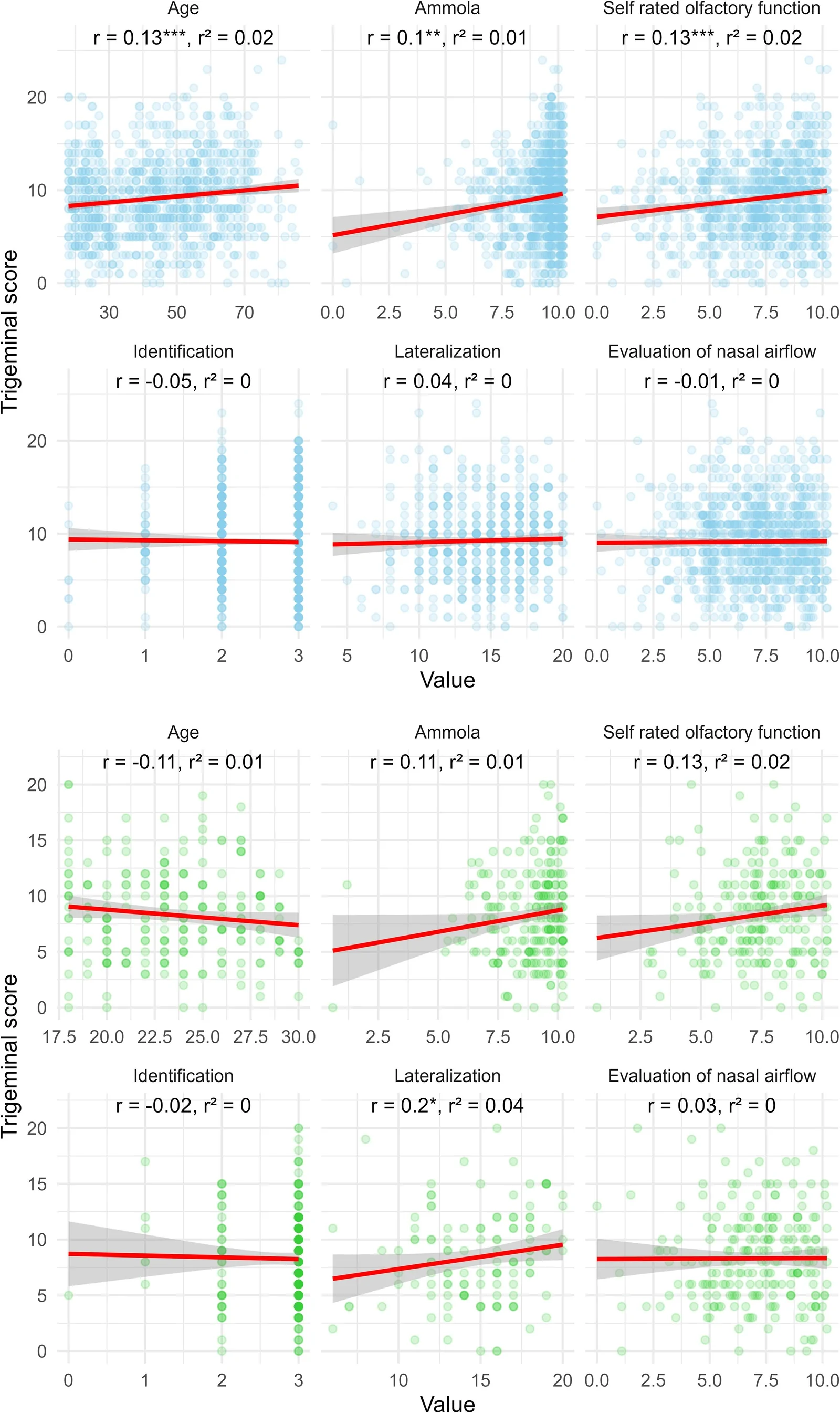
Spearman correlation between trigeminal questionnaire score and age, AmmoLa, identification and lateralization in the general sample (upper half) and the subsample of healthy individuals of 18 to 30 yr (lower half).

To check, whether participants’ score in the new trigeminal questionnaire is coherent with their declarative nose sensitivity, lateralization, identification and AmmoLa scores, correlation analysis was assessed. The results are presented in Table 4 and Fig. 2.

**Table 4:** Spearman's correlation of trigeminal questionnaire scores with lateralization, identification, AmmoLa, age, self-rated olfactory function and self-rating of nasal airflow.

	Trigeminal function questionnaire	Identification	AmmoLa	Lateralization	Nasal sensitivity to stinging/burning	Age	Self-rated olfactory function
Identification	−0.05	…	…	…	…	…	…
AmmoLa	0.10^b^	0.01	…	…	…	…	…
Lateralization	0.04	0.07	−0.04	…	…	…	…
Nasal sensitivity to stinging/burning	0.34^c^	−0.03	0.14^c^	−0.02	…	…	…
Age	0.13^c^	−0.19^b^	0.03	−0.12^b^	0.07^a^	…	…
Self-rated olfactory function	0.13^c^	0.09	0.22^c^	0.05	0.25^c^	−0.05	…
Evaluation of nasal airflow	−0.01	0.05	0.15^c^	0.01	0.08^b^	0.00	0.48^c^

Odor identification and lateralization (*n* = 548, *P* = 0.091), age and evaluation of nasal airflow (*n* = 991, *P* < 0.05).

^a^ *P* < 0.05.

^b^ *P* < 0.01.

^c^ *P* < 0.001.

**Figure 3:**
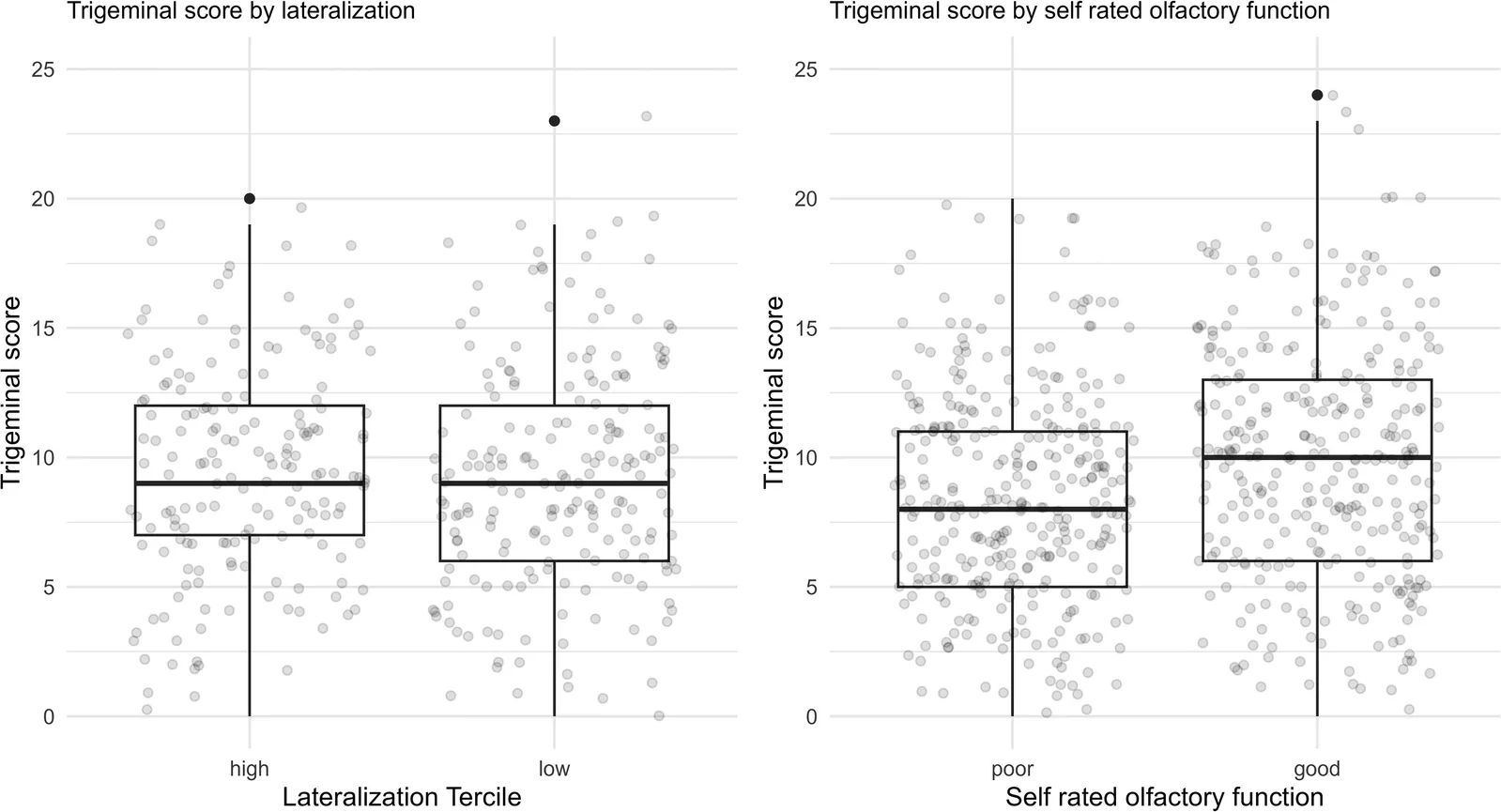
Comparison of trigeminal questionnaire scores across different lateralization and self-rated olfactory function levels. Significant difference was observed in self-rated olfactory function, but not lateralization.

As the nonsignificant correlation of trigeminal questionnaire scores and lateralization scores is counterintuitive, this relation was explored with a different approach—by comparing low and high scores in these 2 abilities, expressed categorically. Lateralization categories were created based on tercile values and scores were grouped accordingly. Participants with high lateralization (*n* = 172) showed slightly higher trigeminal questionnaire scores (*M* = 9.46, *SD* = 4.10) than those low with lateralization (*n* = 200; *M* = 9.28, *SD* = 4.35). This difference was then examined with a *t-*test, showing no significant differences (*t* = −0.42, *P* = 0.68, Cohen's *d* = −0.043). The comparison is showed in Fig. 3.

**Figure 4:**
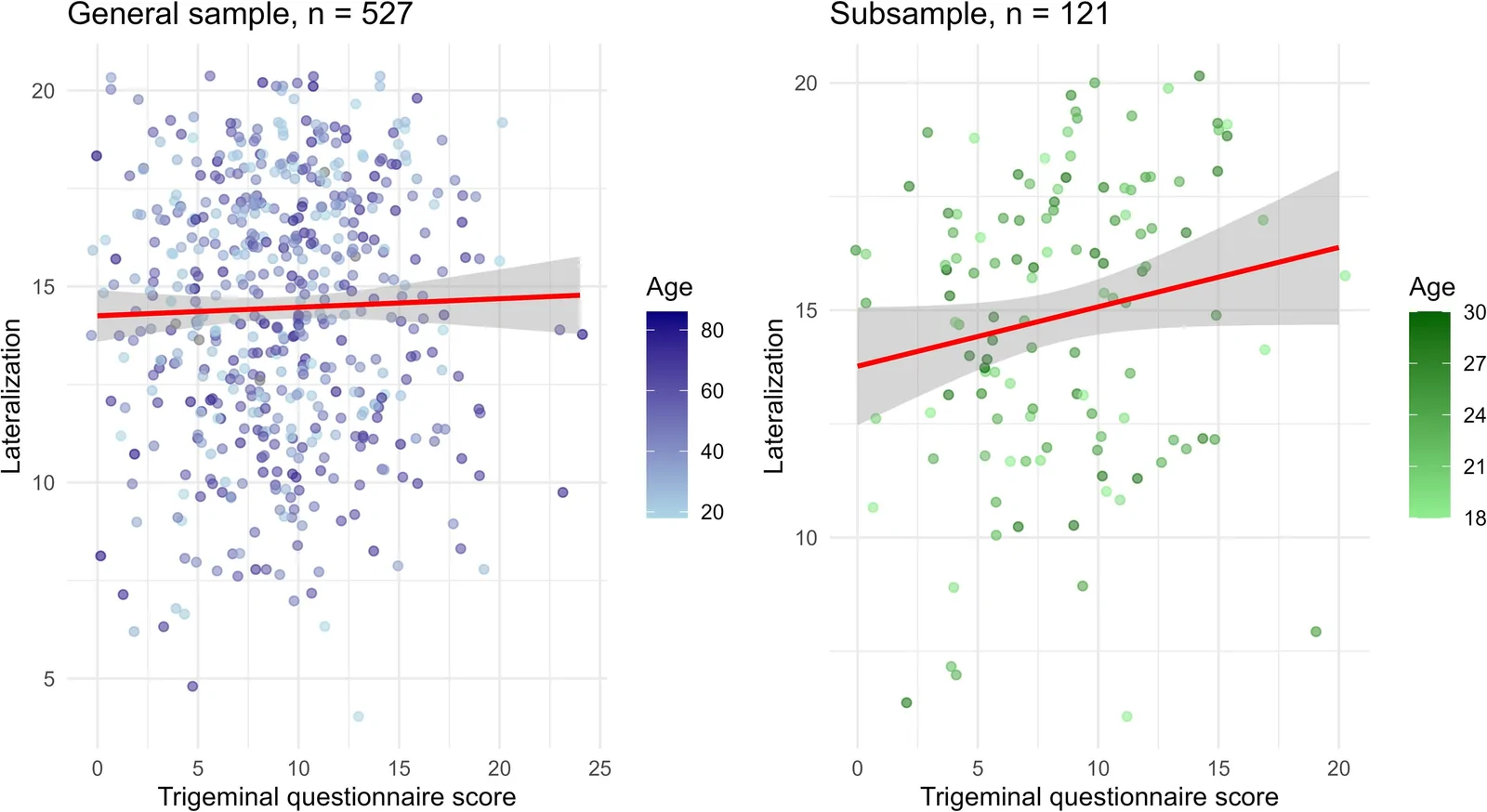
Correlation between trigeminal questionnaire and lateralization in general sample (*n* = 527) and subsample of healthy participants aged 18 to 30 (*n* = 121).

**Figure 5:**
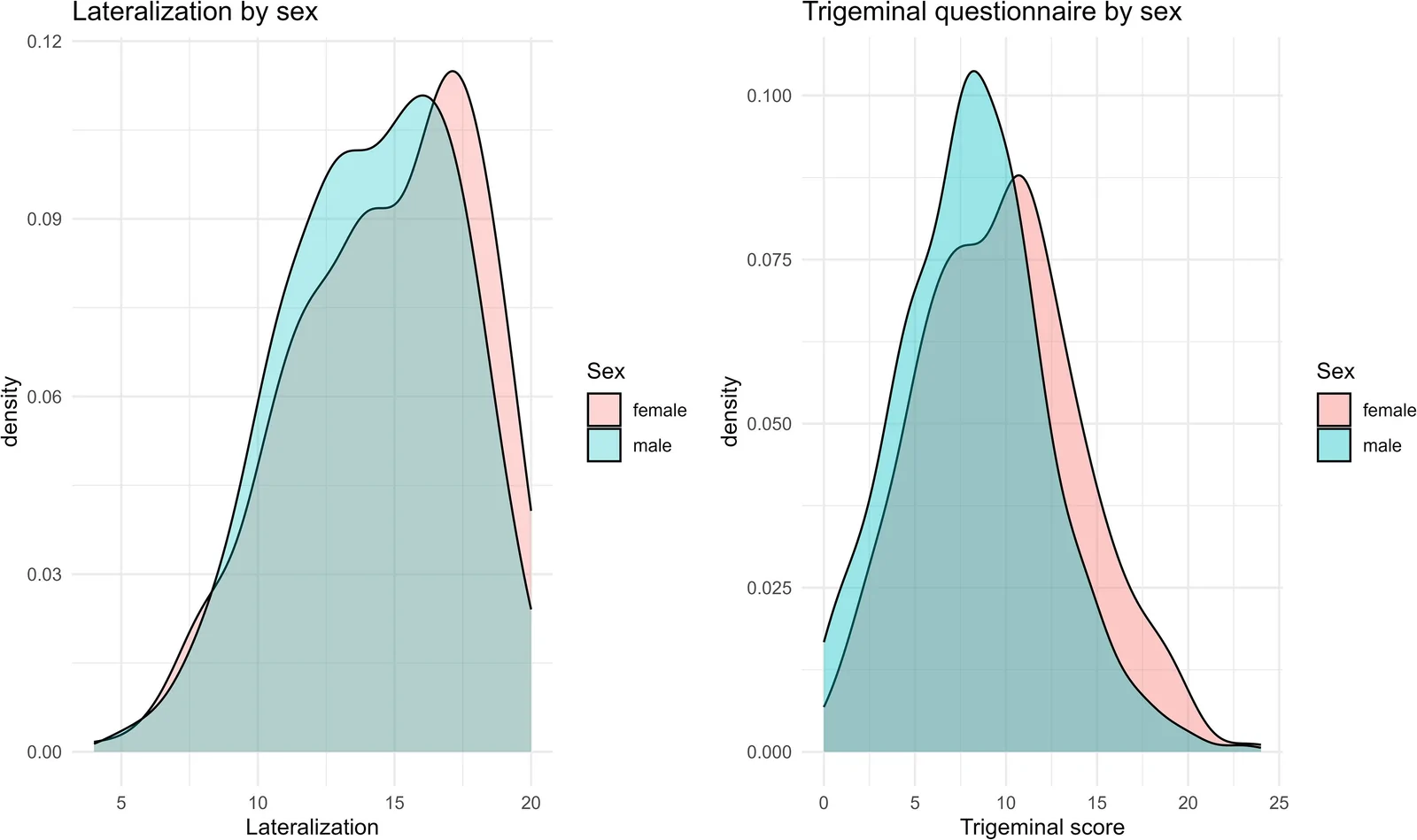
Density of trigeminal questionnaire and lateralization scores among men and women.

As lack of differences in lateralization was not predicted, the distribution of the data was analyzed. As can be read in Fig. 5, the measure seems to be not sensitive enough to differentiate all true levels of participant lateralization—it identifies low lateralization, but high scores are not precisely differentiated. This expected correlation was also explored in a subsample of younger participants (18 to 30 yr old) who did not report any medical conditions (*n* = 121). The correlation was significant in this subsample (*r* = 0.18, *P* < 0.05) which would suggest that in the general sample, the effect may be covered or distracted by age or health related factors. The comparison of correlations between lateralization and trigeminal questionnaire score is provided in scatterplots, in Fig. 4.

The scores in the questionnaire were compared between the sexes. As variances were not equal (Levene's test was significant, *P* < 0.05), nonparametric Welch test was used. There were statistically significant differences among men and women in trigeminal questionnaire score (*P* < 0.001) and AmmoLa (*P* < 0.001). Interestingly, no corresponding difference was found in lateralization (*P* = 0.09), or odor identification (*P* = 0.69). The results of *t*-test are available in Table 5. This divergence raised the possibility that observed differences in questionnaire scores may not reflect true differences in latent factors across genders, but rather arise from gender-specific response pattern. Therefore, a measurement of invariance analysis was conducted to examine this question.

**Table 5:** Results of group comparisons of trigeminal score, AmmoLa, lateralization and identification in men and women, healthy and participants with medical conditions, and COVID-19 recovered and nonpatients.

	*n* (I)	*n* (II)	*M* (I)	*M* (II)	*t*	*P*	Cohen's *d*
**Men (I) and women (II)**							
Trigeminal score	570	387	8.21	9.75	5.63	<0.001	0.37
AmmoLa	569	387	8.91	9.27	4.22	<0.001	0.29
Lateralization	218	311	14.1	14.6	1.73	0.085	0.15
Identification	570	387	2.62	2.64	0.39	0.694	0.03
**Healthy (I) and conditions (II)**							
Trigeminal score	793	170	8.9	10.2	−3.51	<0.001	−0.3
AmmoLa	792	170	9.11	9.21	−0.83	0.405	−0.07
Lateralization	444	88	14.5	14.3	0.20	0.732	0.02
Identification	793	170	2.63	2.60	0.50	0.530	0.04
**Covid (I) and no covid (II)**							
Trigeminal score	797	163	9.00	9.78	−1.99	<0.05	−0.18
AmmoLa	797	192	9.11	9.19	−0.71	0.476	−0.06
Lateralization	436	93	14.6	14.0	1.29	0.200	0.15
Identification	797	163	2.64	2.57	1.24	0.215	0.11

For invariance analysis, 3 models were tested. First the configural model was prepared to check whether model structure of the factor is similar in both groups. The model showed good fit (*CFI* = 0.975, *TLI* = 0.965, *RMSEA* = 0.039), which assured that the basic structure of the questionnaire is the same for women and for men. In the next step, the metric model was prepared to validate if factor loadings are equal across groups. Observed was the minimal change of the fit (*CFI* = 0.975, *TLI* = 0.970, *RMSEA* = 0.036). The *χ*^2^ difference test between the models was not significant, what supported the assumption of equal factor loadings (*χ*^2^ = 6.78, *P* = 0.45). After this, the scalar model was prepared to whether the intercepts are the same in groups of men and women. The fit worsened significantly (*CFI* = 0.936, *TLI* = 0.933, *RMSEA* = 0.054; *χ*^2^ = 52.685, *P* < 0.001), which indicated that intercepts differed across sexes—so differences in questionnaire scores may not necessarily relate to the latent factor itself, but to the approach to responding across genders. In conclusion, the invariance analysis supported configural and metric invariance, but not scalar invariance, implying that factor structure and loadings are consisted across sexes, which permits the use of trigeminal questionnaire for comparing relationships involving the latent factor. However, because of lack of fit of scalar invariance, direct comparison of raw scores between sexes may be slightly biased.

Since scalar invariance was not supported, the score distributions were explored across sexes in more detail. As shown in Fig. 5, the distributions were, in fact, nonadherent. To allow fair and accurate interpretation of individual results, the scores were analyzed separately by sex, then separate normative values were calculated. These are presented in Table 6.

**Table 6:** Mean scores and chosen percentiles of trigeminal questionnaire scores by gender.

	*M* (SD)	*p10*	*p25*	*p50*	*p75*	*p90*
Men	8.21 (4.0)	3	5	8	11	13
Women	9.75 (4.38)	4	7	10	13	16

Since the norms we set differently for men and women, the correlation of questionnaire score with lateralization, AmmoLa, self-rated nasal airflow and self-rated olfactory function was repeated—considering both sexes separately. The results are presented in Table 7. The correlation of trigeminal questionnaire and nasal sensitivity to stinging/burning is present in both men and women, but it is significantly stronger for women. Other correlations did not differ significantly across groups.

**Table 7:** The comparison of correlations of trigeminal questionnaire with olfactory identification, lateralization, AmmoLa, self-rated nasal sensitivity and self-rated olfactory function in men (*n* = 387) and women (*n* = 570) using Fisher's *z* transformation.

	Identification	Lateralization	AmmoLa	Evaluation of nasal airflow	Nasal sensitivity to stinging/burning	Self-rated olfactory function
Trigeminal questionnaire—men	−0.06	−0.05	0.11^a^	0.005	0.27^b^	0.13^a^
Trigeminal questionnaire—women	0.01	0.06	0.11^a^	−0.036	0.41^b^	0.11^a^
Difference (*z*)	1.04	−0.052	−0.035	−0.629	2.38^a^	−0.322

^a^ *P* < 0.05.

^b^ *P* < 0.001.

In the next step, the scores of the questionnaire, olfactory identification, AmmoLa, and lateralization were compared across the group of healthy individuals (*n* = 827) and group of individuals with any reported medical condition (*n* = 174)—including sinusitis, allergies, or chronic rhinosinusitis and others. There was a significant difference in self-reported trigeminal sensitivity across healthy participants and those with medical conditions—healthy participants rated their trigeminal sensitivity as lower than participants with conditions. Results are presented in Table 5.

As the groups of patients and general population is possibly the audience for this questionnaire, invariance was also tested across group of participants with medical conditions and healthy. The procedure was the same as with gender (see above). All models showed good fit (configural *CFI* = 0.967, *TLI* = 0.954, *RMSEA* = 0.045; metric *CFI* = 0.962, *TLI* = 0.954, *RMSEA* = 0.045; and scalar *CFI* = 0.964, *TLI* = 0.962, *RMSEA* = 0.041) and no significant differences in fit were observed—the metric model did not fit significantly worse than the configural model (*χ*^2^(7) = 13.49, *P* = 0.061), and the scalar model did not differ significantly from the metric model either (*χ*^2^(7) = 4.96, *P* = 0.665). The trigeminal sensitivity questionnaire can be therefore considered for using for comparisons of healthy and individuals with medical conditions.

Given that chemosensory abilities are known to decline following a COVID-19 infection, individuals who reported a past COVID-19 infection (*n* = 883) were compared with those who reported never having had COVID-19 (*n* = 165). There was significant difference in self-rated trigeminal sensitivity, but the effect was very small. The results are presented in Table 5.

In the last step, scores of the trigeminal questionnaire were compared between participants who self-reported their olfactory function as either “good” or “poor.” These categories were defined based on tercile values of 1 to 100 ratings. Participants who rated their olfactory function as good (*n* = 324) scored higher in the trigeminal questionnaire (*M* = 9.84), than those, who rated it as poor (*n* = 338, *M* = 8.39). This difference was tested using Welch's *t*-test and found to be statistically significant (*t* = −4.17, *P* < 0.001, Cohen's *d* = −0.33). The comparison is as well showed in Fig. 3.

## 4. Discussion

Based on the study including over 1,000 participants and combining detailed self-report measures with psychophysical assessments, the following main results emerged: (i) From the initial 15-item questionnaire, 8 items were selected as a quick option to rate trigeminal function, yielding good reliability and satisfactory validity. Further subdivision into subscales was not necessary. (ii) Gender differences were observed in the questionnaire of trigeminal function. (iii) The questionnaire of trigeminal function was associated with self-rated olfactory function and nasal airflow, and also correlated with intensity ratings of the AmmoLa.

The aim of the study was to create a self-reporting measure of the sensitivity of the trigeminal function. Although objective, currently available psychophysical measures (such as the lateralization test) are more time-consuming and complex than a simple questionnaire (Garefis et al. 2024). TriFunQ, as a self-assessed, screening method can help fill a diagnostic gap, making the process more efficient and better targeted to those in need. While it does not provide information about the objective sensitivity of trigeminal perception—a limitation discussed below—it does capture the patient's perceived comfort or adequacy of that function, which as well can be a good starting point for the diagnostic process.

A subset of questions was selected that reflects different trigeminal modalities, including thermal, chemical, and nociceptive stimulation. Furthermore, the selected items capture a range of experiential domains related to trigeminal function, addressing cognitive associations, practical application in everyday actions, and consequences of altered perception comparable to the questionnaire of individual significance of olfaction (Croy et al. 2010). This structure allows for a broad coverage of the trigeminal perceptual spectrum.

The initially intended subdivision of the 15-items questionnaire into distinct factors such as association, application and consequence (similar to Questionnaire on the Significance of Olfaction [Croy et al. 2010]), proved unsuitable for the trigeminal questionnaire based on empirical findings. Based on exploratory factor analysis 8 items were selected from the original 15-item pool based on reflecting chemical, nociceptive and thermal trigeminal stimulation, thereby ensuring coverage of distinct sensory pathways within the trigeminal system. While the final item set ensures conceptual breadth, there is a noticeable emphasis on chemical stimuli. Notably, the item on nasal airflow perception was excluded from the final version based on statistical criteria, despite previous evidence suggesting a close association between nasal airflow perception and trigeminal activity (Yan et al. 2023). One of the reasons for this could be that people are not very aware of their trigeminal function—as can be concluded based on factor analysis. In fact, it is the burning or stinging sensation that draws peoples’ attention to their trigeminal function, not the quality of the airflow.

The distribution of items across the experiential subdomains (2 items on consequence, 3 on application, 3 on association), resulting in a relatively even representation of use-oriented aspects of trigeminal perception. The composition of the questionnaire inevitably influences the outcome and interpretation of the self-reported trigeminal function. Therefore, care must be taken in interpreting scores, as weighting toward specific modalities may bias responses. The measure appears to be efficient and can be broadly used to quickly assess people's experience of their trigeminal function. Only comparisons between men and women should be analyzed carefully—as it was observed that these groups present different response pattern (see below).

The questionnaire was internally consistent and presented good reliability. For validation, there were several analyses prepared: correlations (with AmmoLa, odor identification, and lateralization, evaluations of nasal airflow and olfactory function), between group comparisons (of men and women, healthy individuals and those with medical conditions, and people who have had COVID-19 and those who did not), and tercile analysis (of lateralization and self-assessed goodness of olfactory function). Overall, the questionnaire scores were found to correlate positively with other self-rating measures—AmmoLa, evaluation of nasal breathing and self-evaluation of olfactory function—but, in general, not with the objective measures of odor identification and lateralization. Since age and health-related factors could interfere with the relationship between the trigeminal questionnaire and lateralization, an additional analysis was conducted in a subsample of healthy participants under 30 yr of age. In this group, a correlation between trigeminal questionnaire scores and lateralization was observed, suggesting that such a relationship may exist under certain conditions. However, in the general population, this effect appears to be masked by other confounding factors. Also, after limiting the sample, correlations with AmmoLa and self-evaluated olfactory function did not change in strength—they lost statistical significance, but the size of effects did not noticeably change. As for significance—it is a given that larger samples provide more statistically significant results—so not only them, but autonomous effect sizes were taken into consideration.

The relation between lateralization and trigeminal score in general sample was approached differently—trigeminal questionnaire scores were compared in upper and lower tercile of lateralization and self-rated olfactory function—but no differences were found between high and low lateralization. The same was done for self-rated olfactory function. The comparison showed that people who evaluated their olfactory ability as good significantly differed from those who rated it as poor. Taken together, this suggests that the discrepancies with lateralization scores are unlikely to result from shortcomings in the questionnaire itself. This is consistent with other studies indicating that associations between subjective assessments and objective measures are rather weak (Labus et al. 2003; Hummel et al. 2017; Meiselles et al. 2017), and suggest that people may struggle to describe their internal experiences objectively (Wilson and Dunn 2004). Principally personal and subjective self-rating is not detached from other factors that constitute a person's judgement—it is affected by individual factors (including one's personality, motivations, experiences, cognitive patterns, personal points of reference, social comparisons, emotional aspects, expectations about own health, including wishful thinking and the way, that each individual person perceives pain and lack of comfort, or even situational context, such as setting or timing of examination (Bayer et al. 1998; Preis and Kroener-Herwig 2012; Gómez-Pérez and López-Martínez 2013; Peerdeman et al. 2016; Boring et al. 2021; Lunde et al. 2024)). Momentary reflections of 1 aspect of functioning, for example trigeminal sensitivity, probably do not reflect all these factors.

Other tests were involved in group comparisons of trigeminal sensitivity—between healthy individuals and those with health conditions, and between those who recovered from and never had COVID-19 (as the infection affects not only olfactory abilities but also subjective perception of the function after recovery [Vaira et al. 2020]). It was found that higher trigeminal sensitivity was reported by people with reported medical conditions than healthy, and those who never have had COVID-19, than those who recovered. Both these findings are intuitive and align with previous data (as COVID-19 reduces nasal capacity and cooling sensation [Le Bon et al. 2021; Vaira et al. 2022; Azcue et al. 2024]), although lateralization, odor identification and AmmoLa did not differ significantly across those groups. People's tendencies to overestimate their own sensitivity (Meiselles et al. 2017), and underrate their functional recovery after COVID-19 (Cancellieri et al. 2023) may apply here, what could be a potential explanation for the lack of difference in objective measures. Invariance analysis of groups of healthy and nonhealthy participants suggests that the trigeminal sensitivity questionnaire may be useful for comparing these populations. However, in this study the comparison was exploratory and not central to the design. The nonhealthy group was treated as a broad category, without distinguishing between specific clinical backgrounds. Future studies could follow up on this by testing whether people with different types of health conditions differ not only from healthy participants, but also from each other. Differences in trigeminal sensitivity were also shown in men and women with women being more sensitive than men. The invariance analysis showed that while the factor structure and factor loadings of the trigeminal sensitivity questionnaire were consistent across sexes, the intercepts were not. This result requires special attention, as it suggests that observed score differences may reflect group-specific response patterns rather than true differences in the underlying trait. Since the latent trait is, in principle, underlying and cannot be directly measured, it remains unresolved, whether women overestimate or men underestimate their sensitivity. Additionally, in the matter of sensitivity and pain gender and gender roles may affect self-assessment (Jones and Zachariae 2002; Myers et al. 2003; Fillingim et al. 2009). Hence, separate norms for self-rated trigeminal sensitivity were calculated for men and women.

Significantly different scores were also obtained in a group of healthy participants and individuals with medical conditions. The invariance analysis was again assessed and showed that all models fit good. This justifies the use of the trigeminal questionnaire in both groups, and between group comparisons as well. Healthy individuals reported less trigeminal sensitivity than people with any kind of reported medical condition. Again, there were no significant differences in psychophysical measures of odor identification and lateralization—suggesting that the 2 groups did not differ widely in sensitivity, but more in terms of the participant's attention to the sensation, or just the subjective pain rating.

Overall, people do not appear to be precise at self-rating objective pain level and internal, physiological states (Ahluwalia et al. 2020; Baroni et al. 2022)—as studies show, self-assessment and observed pain behavior are only moderately correlated (Labus et al. 2003). A similar issue was observed in our study. One explanation is that people lack precise introspective access to their physiological states, hence we should not expect strong correlations between self-report scales and objective measurements, at least in physiological matter. The limit of self-assessment is that subjective reports will often diverge from physiological signals—and are open to individual interpretation. This is the other explanation—in questionnaires like this people do not try to assess their objective physiological state compared with that of other people but also evaluate individual experiences relating to this state. The same objectively measured trigeminal sensitivity possibly may bring an almost neutral sensation for 1, discomfort or irritation for the next, and pain to the other—as experiences are, as the matter of principle, subjective. As this limitation is rooted in the nature of introspection and shape of cognitive and emotional factors (Tracey and Mantyh 2007), these discrepancies are generally present. The lack of correlation with objective measures does not mean self-reporting measures are useless. On the contrary—they offer valuable insights into how people perceive their own states, and feel about their health and organism which is of high diagnostic and prognostic significance.

The present validation was conducted in a population recruited in Dresden, Germany, and the questionnaire was administered in German. Although the items refer to common trigeminal sensations encountered in everyday life (e.g. pungent odors, menthol, cold air, or carbonation), the psychometric properties of the questionnaire have so far been established only in this cultural and linguistic context. Therefore, formal cross-cultural adaptation and validation will be necessary before the instrument can be confidently applied in other populations. In addition, ethnic or racial background of participants was not systematically recorded in the present study. As recruitment took place in a public museum in Dresden, the sample likely reflects the demographic composition of the local population, which is predominantly of European background, but this cannot be confirmed based on the collected data. Future studies should therefore evaluate the questionnaire in more culturally and demographically diverse samples.

Objective score and subjective sensation should not be forced and bound in 1 index, but understood as 2 separate issues. One brings the knowledge on the patient's physiology, and the other reveals if the person is satisfied and comfortable with the way their organism functions. Both issues should be addressed with an adequate methodology.

This questionnaire constitutes an important first step toward the structured assessment of trigeminal function. Its application in larger cohorts of both healthy individuals and patients with olfactory and/or trigeminal dysfunction is crucial to further establish its psychometric validity and explore its clinical relevance. Broader implementation will be necessary to fully determine its diagnostic potential and support the development of standardized protocols and normative reference data.

## 5. Conclusion

The 8-item TriFunQ demonstrated good reliability and satisfactory validity. The questionnaire scores correlate positively with the intensity ratings for AmmoLa and self-reporting assessment of olfactory function and nasal airflow, indicating consistency in participants’ subjective assessment of their trigeminal function. Lateralization, included as an objective indicator, was associated with the scores only in a subsample of healthy individuals aged 30 or younger. A 3-factor structure was explored but did not find empirical confirmation. Separate normative data are recommended for men and women due to sex differences. These findings support the use of the TriFunQ as a brief and reliable tool for assessment of self-perceived trigeminal function.

## Supplementary Material

bjag013_Supplementary_Data

## Data Availability

R analyses are available: https://github.com/wiktoriajedryczka/R-scripts/blob/6ec0b6e734471db1781ab92783d9388e41fe75a1/Trigeminal_tidyversion.R.
