# Correction to: The trigeminal function questionnaire (TriFunQ): a tool for clinical and research use

**DOI:** 10.1093/chemse/bjag030

**Published:** 2026-10-19

This is a correction to: Wiktoria Jędryczka, Susanne Weise, Anabell Bormann, Thomas Hummel, The trigeminal function questionnaire (TriFunQ): a tool for clinical and research use, *Chemical Senses*, Volume 51, 2026, bjag013, https://doi.org/10.1093/chemse/bjag013

In the originally published version of this manuscript, the ready-to-print 8- and 15-question versions of the TriFunQ questionnaire were omitted.

They have now been added as supplementary material.

