# Supplementary material for: The trigeminal function questionnaire (TriFunQ): a tool for clinical and research use
Source: Chem Senses. 2026 May 14;51:bjag013. doi: 10.1093/chemse/bjag013 (PMC13198209; doi:10.1093/chemse/bjag013)
Supplement: Supplementary file 1 [file bjag013_supplementary_data.pdf]

The Trigeminal Function Questionnaire (TriFunQ)- 8 item

The following questionnaire concerns the impact of your trigeminal system on everyday experiences. Please answer all questions based on your immediate impression, as there are no right or wrong responses.

	I totally agree	I rather agree	I rather disagree	I totally disagree
I avoid carbonated drinks because you feel a stinging sensation in your nose when you burp.				
Pungent or burning smells make me cough or sneeze.				
I avoid burning, pungent smells (e.g. ammonia or chlorine).				
When I smell something pungent or stinging, I panic and think of situations in which something similar has happened to me.				
In winter, the cold air in my nose is extremely unpleasant.				
When I eat horseradish, I find the stinging in my nose particularly annoying.				
Burning or pungent smells can cause unpleasant sensations/pain in my face.				
When it comes to slightly tickly or pungent smells, my nose is much more sensitive than other people's noses.				

The Trigeminal Function Questionnaire (TriFunQ)- 15 item

The following questionnaire concerns the impact of your trigeminal system on everyday experiences. Please answer all questions based on your immediate impression, as there are no right or wrong responses.

	I totally agree	I rather agree	I rather disagree	I totally disagree
Pungent or burning smells like smoke, vinegar or nail polish remover evoke strong emotions in me.				
When I chew a fresh mint gum, I feel like I can breathe better through my nose.				
I avoid carbonated drinks because you feel a stinging sensation in your nose when you burp.				
I consider my nasal breathing to be very good.				
I don't like going to a sauna because I find hot air in my nose very burning.				
When I cut onions, my eyes water a lot.				
Pungent or burning smells make me cough or sneeze.				
I avoid burning, pungent smells (e.g. ammonia or chlorine).				
When I smell something pungent or stinging, I panic and think of situations in which something similar has happened to me.				
In winter, the cold air in my nose is extremely unpleasant.				
When I eat horseradish, I find the stinging in my nose particularly annoying.				
Burning or pungent smells can cause unpleasant sensations/pain in my face.				
I am aware and intensely aware of the carbonation in drinks.				
When it comes to slightly tickly or pungent smells, my nose is much more sensitive than other people's noses.				
I only use toothpaste with a very mild mint smell.				
